# Ginsenoside Rg3 combined with near‐infrared photothermal reversal of multidrug resistance in breast cancer MCF‐7/ADR cells

**DOI:** 10.1002/fsn3.4205

**Published:** 2024-05-21

**Authors:** Ying Chang, Qiang Fu, Zhongqi Lu, Quanxin Jin, Tiefeng Jin, Meihua Zhang

**Affiliations:** ^1^ Department of Ultrasound Medicine Affiliated Hospital of Yanbian University Yanji China; ^2^ Department of Pathology and Cancer Research Center Yanbian University Medical College Yanji China; ^3^ Key Laboratory of the Science and Technology, Department of Jilin Province Yanji China; ^4^ Department of Immunology and Pathogenic Biology Yanbian University Medical College Yanji China

**Keywords:** adriamycin, ginsenoside Rg3, near‐infrared photothermal therapy, reversing drug resistance

## Abstract

Adriamycin (ADR) is a frequently employed chemotherapeutic agent for the management of breast cancer. Nevertheless, multidrug resistance (MDR) can impair its therapeutic efficacy in breast cancer. MDR is characterized by increased expression of the P‐glycoprotein (P‐gp) efflux pump, up‐regulation of anti‐apoptotic proteins, and downregulation of pro‐apoptotic proteins. Consequently, inhibition of ATP‐binding cassette (ABC) transporter proteins has been deemed the most efficacious approach to overcome MDR. In this study, we used MTT (3‐[4,5‐dimethylthiazol‐2‐yl]‐2,5 diphenyl tetrazolium bromide), Western blots, flow cytometry, immunofluorescence, and constructed xenograft tumors to investigate whether ginsenoside Rg3‐near‐infrared photothermal (Rg3‐NIR) combination reversed multidrug resistance in MCF‐7/ADR breast cancer. In vivo and in vitro experiments, the results showed that Rg3‐NIR co‐treatment was effective in inducing the apoptosis of MCF‐7/ADR breast cancer cells. This was achieved by reversing the expression of drug resistance‐associated proteins, while also inhibiting cell proliferation, migration, and epithelial–mesenchymal transition (EMT) processes via attenuation of the phosphatidylinositol 3‐kinase (PI3K)/protein kinase B (AKT) signaling pathway transduction. Ginsenoside Rg3 combined with near‐infrared photothermal therapy (NIR) effectively reverses multidrug resistance in breast cancer MCF‐7/ADR cells, providing a new therapeutic strategy for breast cancer drug resistance.

## Graphical abstract

In consequence, the ginsenoside Rg3–near‐infrared photothermal therapy (Rg3–NIR) combination treatment group could inhibit MCF‐7/ADR cell migration, invasion, angiogenesis, and epithelial–mesenchymal transition (EMT) process by inhibiting the phosphatidylinositol 3‐kinase protein kinase B–mammalian target of rapamycin (PI3K‐AKT–mTOR) signaling pathway, reverse the expression of drug resistance‐related proteins, such as ATP‐binding cassette subfamily G member 2 (ABCG2) and multidrug resistance (MDR), and also promote the increase of apoptosis in MCF‐7/ADR cells, and reverse the drug resistance of MCF‐7/ADR cells.

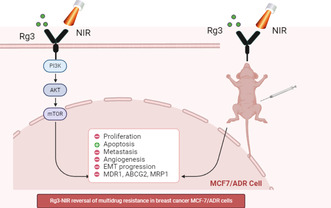

## 1 INTRODUCTION

In 2023, after the report “Estimates of Global Cancer Incidence and Mortality” was published, breast cancer has become the most prevalent malignant tumor among women worldwide (Goff & Danforth, 2021; Siegel et al., 2023; Sung et al., 2021). The current modalities utilized in the management of breast cancer comprise surgical intervention, radiation therapy, cytotoxic chemotherapy, hormonal manipulation, and targeted therapy (Dragojevic et al., 2022; So et al., 2022). The emergence of multidrug resistance (MDR) stands as the primary cause of chemotherapy ineffectiveness (An et al., 2021; Boichuk et al., 2022; Long et al., 2022). MDR continues to pose a significant challenge in cancer therapy, displaying distinct features, such as the overexpression of the P‐glycoprotein (P‐gp) efflux pump, upregulation of anti‐apoptotic proteins, and downregulation of pro‐apoptotic proteins. In addition, intrinsic or acquired resistance of breast cancer to chemotherapeutic agents, tumor heterogeneity, contributes to the development of resistance. Numerous studies have elucidated the integral role of P‐glycoprotein (P‐gp), breast cancer resistance protein (BCRP) (Hanker et al., 2020; Zhang et al., 2020; Zhang, Li, et al., 2022; Zhang, Yang, et al., 2022), and MDR‐related protein as members of the ATP‐binding cassette (ABC) transporter family, with their upregulated expression being a pivotal factor contributing to MDR (Groza et al., 2020), and drug resistance being a big obstacle to obtain the desired clinical prognosis (Xing et al., 2023). The intricate mechanisms underlying resistance underscore the imperative for extensive research and the advancement of innovative therapeutic approaches aimed at surmounting chemotherapy resistance in breast cancer.

Ginsenoside Rg3, a herbal medicine derived from the root of *Panax ginseng*, has shown promise in the treatment of breast cancer. It manifests diverse pharmacological properties, encompassing anti‐inflammatory, antioxidant, and antiproliferative effects, which may aid in impeding the proliferation and metastasis of breast cancer cells (Wang et al., 2023; Xia, Ma, et al., 2022; Xia, Zhang, et al., 2022). Meanwhile, Ginsenoside has been found to induce apoptosis (programmed cell death) and inhibit angiogenesis (Zeng et al., 2022). Moreover, it has shown potential in modulating hormone receptors and signaling pathways involved in breast cancer development (Li et al., 2017; Zhang et al., 2019). It has been shown that red ginsenoside Rg3‐based liposomes loaded with paclitaxel treat cancer drug resistance by dual targeting of the tumor microenvironment and cancer cells (Zhu et al., 2023).

Near‐infrared photothermal therapy (NIR) has received extensive attention due to its high efficiency and specificity (Wei et al., 2022). Through the utilization of photothermal conversion agents, light irradiation can be absorbed and transformed into thermal energy, facilitating the destruction of tumor tissue while minimizing adverse effects. The optimal photothermal conversion agent should possess nontoxic characteristics, exhibit high tumor specificity, and demonstrate efficient conversion of light into heat (Zhou et al., 2021). Several studies have demonstrated that NIR can benefit patients with recurrent cancer (Datta et al., 2016; Overgaard et al., 2009). The controllably generated nitric oxide (NO) under NIR irradiation can effectively reverse multidrug resistance by inhibiting the overexpression of P‐gp and cell respiration, significantly enhancing the concentration of chemotherapeutic agents in tumor cells (Wei et al., 2019).

Adriamycin (ADR), an anthracycline chemotherapy drug used in breast cancer treatment, exerts its effects through multiple mechanisms. It intercalates into the DNA double helix, inhibiting topoisomerase II and causing DNA damage (Li et al., 2022). It generates reactive oxygen species (ROS), leading to oxidative stress and apoptosis. Doxorubicin disrupts cell membrane integrity, affects protein synthesis, and activates apoptotic pathways. These actions collectively inhibit cancer cell growth and promote cancer cell death, making doxorubicin an effective treatment option for breast cancer. Its mechanism of action involves inhibition of the phosphatidylinositol 3‐kinase/protein kinase B (PI3K/AKT) signaling pathway, which plays a critical role in breast cancer progression (Yu et al., 2021; Zhong et al., 2022). However, prolonged use of ADR can lead to drug resistance. In this study, we investigated the efficacy of Rg3 and NIR in reversing MDR in MCF‐7/ADR breast cancer cells. Mechanistic investigations were conducted to elucidate the underlying mechanisms of drug resistance reversal in human breast cancer.

## 2 MATERIALS AND METHODS

### 2.1 Cell lines

The Yanbian University Cancer Research Center (Yanji, China) supplied the human breast cancer cell line MCF‐7 and MCF‐7/ADR were obtained from iCell Bioscience Inc. (Shanghai, China). To sustain the multidrug resistance phenotype, the culture medium of the MCF‐7/ADR cell line was supplemented with 0.5 μg/mL of ADR.

### 2.2 Near‐infrared photothermal therapy

A 750 W halogen lamp and a 780 nm high‐pass filter were used to heat water‐filtered infrared A (wIRA) (Hydrosun 750 model, Hydrosun Medizintechnik GmbH, Müllheim, Germany), with an output peak at 820 nm. The near‐infrared device was preheated to 37, 40, 43, and 46°C, and then applied to culture dishes for 3, 5, and 10 min, respectively.

### 2.3 MTT (3‐[4,5‐dimethylthiazol‐2‐yl]‐2,5 diphenyl tetrazolium bromide) assay

The cells were seeded at a density of 1.0 × 10^4^ cells per well in a 96‐well plate. Subsequently, the cells were treated with varying concentrations of ADR (10, 20, 40, 80, 160, 320, 640, 1280 and 2560 ng/mL) and Rg3 (at concentrations of 50, 100, 150, 200 μg/mL) for 24, 48, and 72 h. Following each time point, 80 μL of culture medium and 20 μL of MTT were added to each well. After this, viable cells were quantified by measuring the absorbance at 490 nm following solubilization of the formed crystals with dimethylsulfoxide (DMSO).

### 2.4 Colony formation assay

The MCF‐7/ADR cells were seeded in 6‐well plates at a density of 500 cells per well and allowed to adhere for 24 h. Following this, the cells were treated with varying concentrations of Rg3, NIR, or Rg3–NIR for a duration of 48 h. Subsequently, the cells were cultured for an additional 14 days. At the end of the culture period, the cells were fixed with methanol, washed using phosphate‐buffered saline (PBS), and stained with Giemsa. The number of colonies formed was quantified as a measure of cell proliferation.

### 2.5 Hoechst 33342 assay

Cells were cultured on coverslips placed in 6‐well plates and subsequently fixed using 4% paraformaldehyde. Following fixation, the cells were permeabilized with 0.5% Triton X‐100 and blocked with 3% bovine serum albumin (BSA). Hoechst 33342 was applied to the cells for staining.

### 2.6 Annexin V–propidium iodide (PI) analyzed by flow cytometry

The MCF‐7/ADR cell lines, each consisting of 1 × 10^6^ cells, underwent two washes with binding buffer, followed by staining with the Annexin V‐FITC (Fluorescein Isothiocyanate) Apoptosis Detection Kit. Subsequently, the stained cells were meticulously analyzed utilizing a BD Accuri C6 Flow Cytometer, and the resulting data were meticulously processed using the sophisticated FlowJo V10.5.3 software, ensuring precise and comprehensive analysis of apoptotic events at the single‐cell level.

### 2.7 Western blot

MCF‐7/ADR cells were carefully harvested and the total protein extraction was performed using radioimmunoprecipitation assay (RIPA) lysate. The proteins were carefully separated using sodium dodecyl sulfate‐polyacrylamide gel electrophoresis (SDS–PAGE) analysis, and the separated proteins were transferred to polyvinylidene fluoride (PVDF) membranes, which were blocked with skimmed milk for 1 h to prevent nonspecific binding, and then the membranes were incubated with primary antibody. Antibody signals were meticulously detected using an advanced chemiluminescence system for comprehensive and reliable quantitative analysis.

### 2.8 Wound healing assay

MCF‐7/ADR breast cancer cells were cultured in 6‐well plates until reaching an appropriate growth status. Subsequently, various concentrations of Rg3, NIR, or Rg3–NIR were administered to the cells for a duration of 48 h. To assess the migratory potential of the cells, scratch wounds were created using a 200 μL pipette tip. Microscopy images were acquired at 0, 24, and 48 h posttreatment to evaluate the healing process.

### 2.9 Cell migration assay

MCF‐7/ADR cells were exposed to varying concentrations of Rg3, NIR, or Rg3–NIR for a duration of 48 h. Following digestion, 5 × 10^4^ cells were seeded into the upper chamber of Transwell devices. The chambers were separated by 8‐μm pore size membranes in serum‐free Dulbecco's Modified Eagle Medium (DMEM). Subsequently, the membranes were fixed with paraformaldehyde and stained with hematoxylin. Enumeration of migrated cells was performed through microscopic examination.

### 2.10 Endothelial cell tube formation assay

After thoroughly mixing microliters of Matrigel and serum‐free medium at an exact ratio of 1:1, 60 μL of the mixture was subsequently carefully added to each well of a 96‐well plate, ensuring that air bubbles were avoided, and then incubated for 4 h. Digest and process cells with different concentrations of Rg3, NIR alone or in combination for 48 h, resuspend them in conditioned media at a volume of 1 × 10^4^ cells/150 μL, and inoculate them in wells containing Matrigel. After a further 6 h of incubation, the cell network was carefully observed under a microscope and photographed using precise photographic techniques.

### 2.11 Immunofluorescence (IF)

For immunostaining, cells were cultured on (24 × 24 mm) slides, washed with PBS, fixed with 2% paraformaldehyde for 30 min, permeabilized with 0.5% Triton X‐100 (CWBIO, Beijing, China), sealed with BSA (Solarbio, Beijing, China), and incubated with primary antibodies at 4°C overnight, then followed by Alexa Fluor 568 Goat Anti‐mouse immunoglobulin G (IgG) (H + L) secondary antibody. Sections were sealed with an antifading fluorescent fixator containing 4′,6‐diamino‐2‐phenylindole (DAPI, Solarbio, Beijing, China). The intensity of fluorescence image was observed and photographed under a microscope.

### 2.12 In vivo tumorigenesis assay

Fifteen female BALB/c mice, aged 5 weeks and weighing approximately 20 g, were thoughtfully sourced from Beijing Vital River Laboratory Animal Technology Co., Ltd., ensuring the use of high‐quality experimental subjects. These mice were meticulously housed in a controlled environment, maintaining a pathogen‐free facility at a temperature of 22°C with 50% humidity and a 12 h light/dark cycle, to provide optimal conditions for their well‐being. For the experimental procedures, 1 × 10^6^ MCF‐7/ADR cells were delicately mixed with Matrigel and intraperitoneally injected into the right fat pad of the fourth mammary gland of nude mice. Once the average tumor volume of the tumor‐bearing mice reached 150–200 mm^3^, the mice were thoughtfully and randomly assigned to one of the five experimental groups: the saline group (control group) (*n* = 3); the ADR group (*n* = 3); the NIR group (*n* = 3); the Rg3 group (*n* = 3); and the Rg3–NIR group (*n* = 3).

Next, the mice were treated with 6 mg/kg/100 μL (PBS containing) Rg3 once every day. When the mean tumor volume was 150–200 mm^3^, a circular mask with a diameter of 1.5 centimeters was placed on the abdomen to limit radiation to the tumor. Subsequently, intraperitoneal injection of ginsenoside Rg3 was administered, followed by in vivo near‐infrared radiation (1.5 W/cm^2^, 3 min). Tumor volume was meticulously measured using the modified ellipsoid volume formula (volume = 1/2 [length × width^2^]). It is important to note that all procedures involving animals were conducted under the supervision and approval of the Animal Ethics Research Organization of Yanbian University.

### 2.13 Statistical analyses

Statistical analyses were conducted using GraphPad Prism 8.0 software, and the appropriateness and reliability of the data analysis methods were ensured by performing either one‐way analysis of variance (ANOVA) or *t*‐tests. A *p*‐value < .05 indicated statistical significance. A value of *p* > .05 was nonsignificant (ns). a: *p* < .01 versus control group, b: *p* < .01 versus ADR group, c: *p* < .01 versus NIR group, and d: *p* < .01 versus Rg3 group, ****: *P* < .0001 versus control group. All experiments were repeated three times.

## 3 RESULTS

### 3.1 Rg3–NIR inhibits the proliferation of MCF‐7/ADR breast cancer cells

**FIGURE 1:**
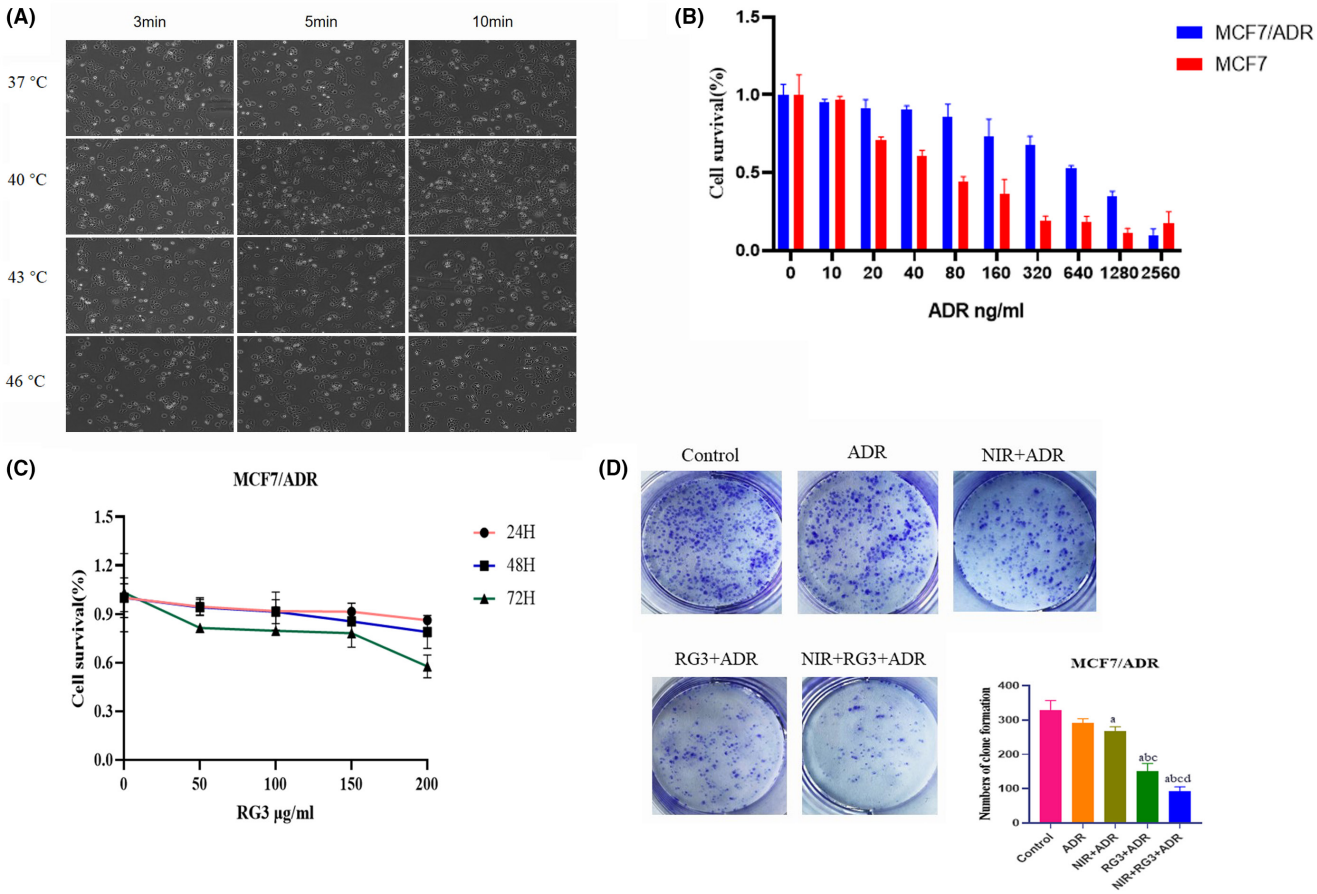
Rg3–NIR inhibits the proliferation of MCF‐7/ADR breast cancer cells. (A) The effects of different near‐infrared photothermal treatments on MCF‐7/ADR breast cancer cells. (B) The MTT assay was performed to detect the effect of ADR on MCF‐7 cells and MCF‐7/ADR cell activity. (C) Cytotoxicity of Rg3 treatment on MCF‐7/ADR cells after 24–72 h was determined using the MTT assay. (D) Effect of RG3 (50 μg/mL) and NIR (43°C, 3 min) by colony formation assay. a: *p* < .01 versus control group, b: *p* < .01 versus ADR group, c: *p* < .01 versus NIR group, d: *p* < .01 versus Rg3 group.

In order to investigate the impact of various NIR on MCF‐7/ADR breast cancer cells, we conducted a series of experiments, using NIR, to treat MCF‐7/ADR breast cancer cells at 37, 40, 43, and 46°C for 3, 5, and 10 min (Figure 1A). These experiments were conducted based on the observed tendency of breast cancer cells to exhibit retraction, rounding, decreased size, reduced light transmittance, and increased floating. Following a comprehensive assessment of the cellular growth dynamics, the temperature and duration for NIR exposure were meticulously established at 43°C for a duration of 3 min.

To evaluate the inhibitory effects of ADR on the proliferation of MCF‐7 and MCF‐7/ADR breast cancer cells, as well as their respective drug resistance, we used the MTT assay. Different concentrations of ADR (10, 20, 40, 80, 160, 320, 640, 1280 and 2560 ng/mL) were administered to the breast cancer cells for varying durations of 24, 48, and 72 h (Figure 1B). As the dosage increased, we observed a gradual decrease in the proliferation rate of MCF‐7 cells. In contrast, the survival rate of MCF‐7/ADR cells demonstrated no significant difference. These findings unequivocally highlight the inherent resistance of MCF‐7/ADR cells to ADR, despite increasing exposure. Notably, the IC50 values, representing the concentration of ADR required to inhibit cell growth by 50%, were determined as 47.46 ng/mL for MCF‐7 cells and 1040 ng/mL for MCF‐7/ADR cells, respectively. This substantial difference underscores the heightened resistance of MCF‐7/ADR cells to ADR treatment. The resistance index (RI) was 21.92. This notable disparity underscores the pronounced resistance of MCF‐7/ADR cells to the inhibitory effects of ADR. In the follow‐up experiments, MCF‐7/ADR cells were cultured with ADR drug concentration culture medium containing 500 ng/mL.

Similarly, the inhibitory ability of ginsenoside Rg3 monotherapy on MCF‐7/ADR cells was examined using MTT. Using Rg3 (50, 100, 150, and 200 μg/mL) in 24, 48, and 72 h, respectively, the 90% maximal effective concentration (EC90) value of Rg3 on MCF‐7/ADR cells was 50 μg/mL, and the concentration of Rg3 was chosen subsequently (Figure 1C).

To assess the potential synergistic effect of Rg3–NIR in reversing activity in MCF‐7/ADR cells, we conducted a colony formation assay. Following the previous results, cells were treated with Rg3 (50 μg/mL) and NIR (43°C, 3 min). The findings revealed that the combined treatment of Rg3 and NIR exhibited the most potent inhibitory effect on colony formation (Figure 1D).

### 3.2 Rg3–NIR promotes apoptosis of MCF‐7/ADR breast cancer cells

**FIGURE 2:**
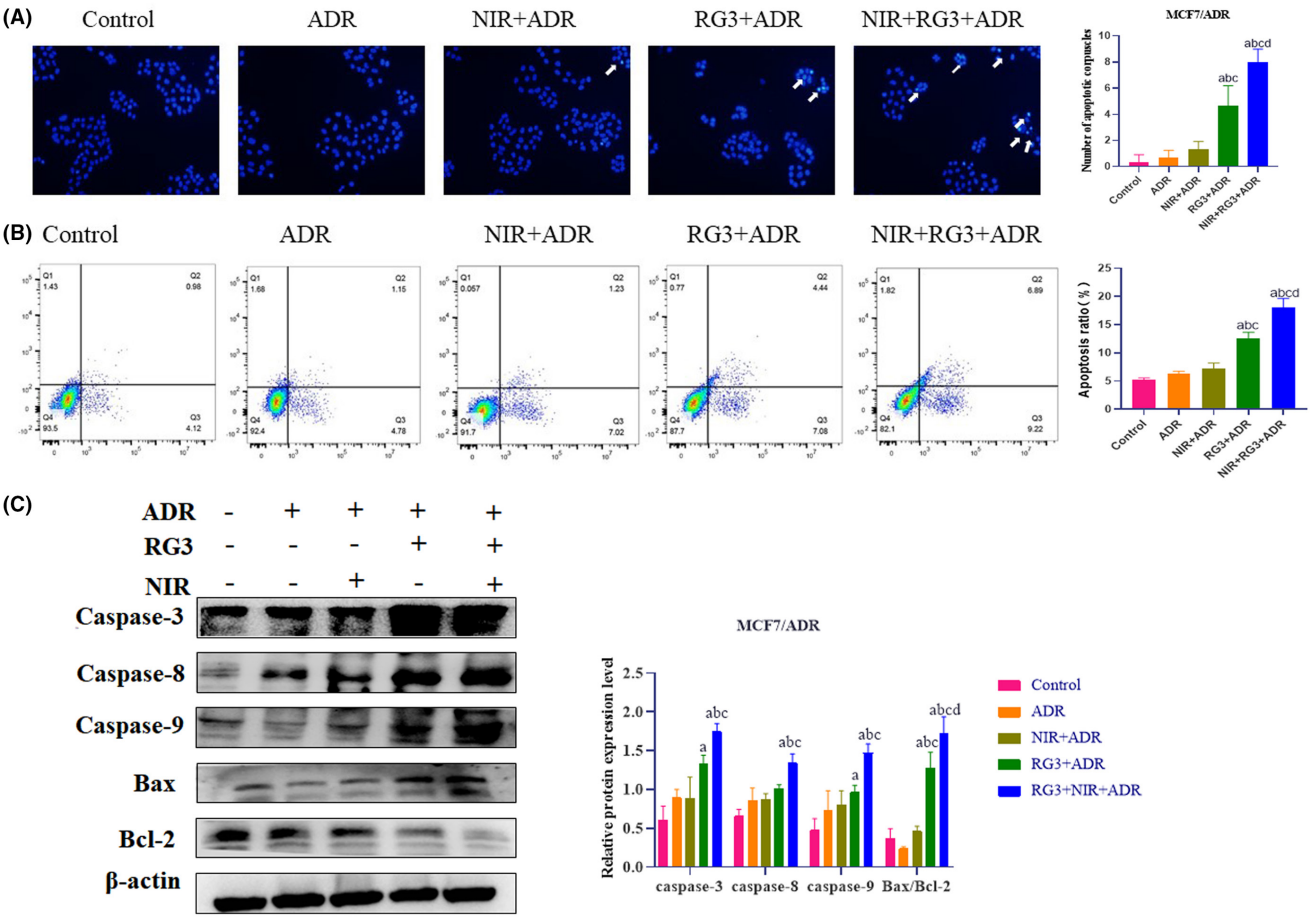
Near‐infrared photothermal therapy combined with ginsenoside Rg3 promotes apoptosis of MCF‐7/ADR breast cancer cells. (A) Number of apoptotic corpuscles of MCF‐7/ADR cells induced by different treatment groups after 48 h of incubation determined using the Hoechst 33342 stain assay (200×). (B) Apoptosis of MCF‐7/ADR cells induced by different treatment groups after 48 h of incubation determined using the Annexin V‐FITC/PI staining. (C) The levels of apoptosis‐related proteins in MCF‐7/ADR breast cancer cells after different treatment groups. a: *p* < .01 versus control group, b: *p* < .01 versus ADR group, c: *p* < .01 versus NIR group, d: *p* < .01 versus Rg3 group.

As our previous research, in Hoechst 33342 stain assay (Figure 2A), the ADR and NIR groups had no effect on cell apoptotic vesicle production, Rg3 groups could increase the production of apoptotic bodies in MCF‐7/ADR cells, but more apoptotic vesicles were produced in the Rg3–NIR group. The increased apoptosis rate was reflected in the flow cytometry assay (Figure 2B). Compared with the control group, ADR and NIR groups had no effect on cell apoptosis, but the apoptosis rate increased after 48 h of treatment in the Rg3 group and the Rg3–NIR group.

To elucidate the mechanism by which Rg3–NIR augments the pro‐apoptotic activity of ADR, we examined the expression levels of relevant proteins via Western blot analysis (Figure 2C). The Rg3–NIR resulted in significantly increased expression of the pro‐apoptotic protein (Bax [Bcl‐2‐associated X‐protein]) and a concomitant decrease in the expression of the anti‐apoptotic protein (Bcl‐2 [B‐cell lymphoma protein 2]). These findings suggest that the Rg3–NIR treatment enhances apoptosis in MCF‐7/ADR cells by upregulating pro‐apoptotic proteins and downregulating anti‐apoptotic proteins.

### 3.3 Rg3–NIR inhibits MCF‐7/ADR breast cancer cell metastasis

**FIGURE 3:**
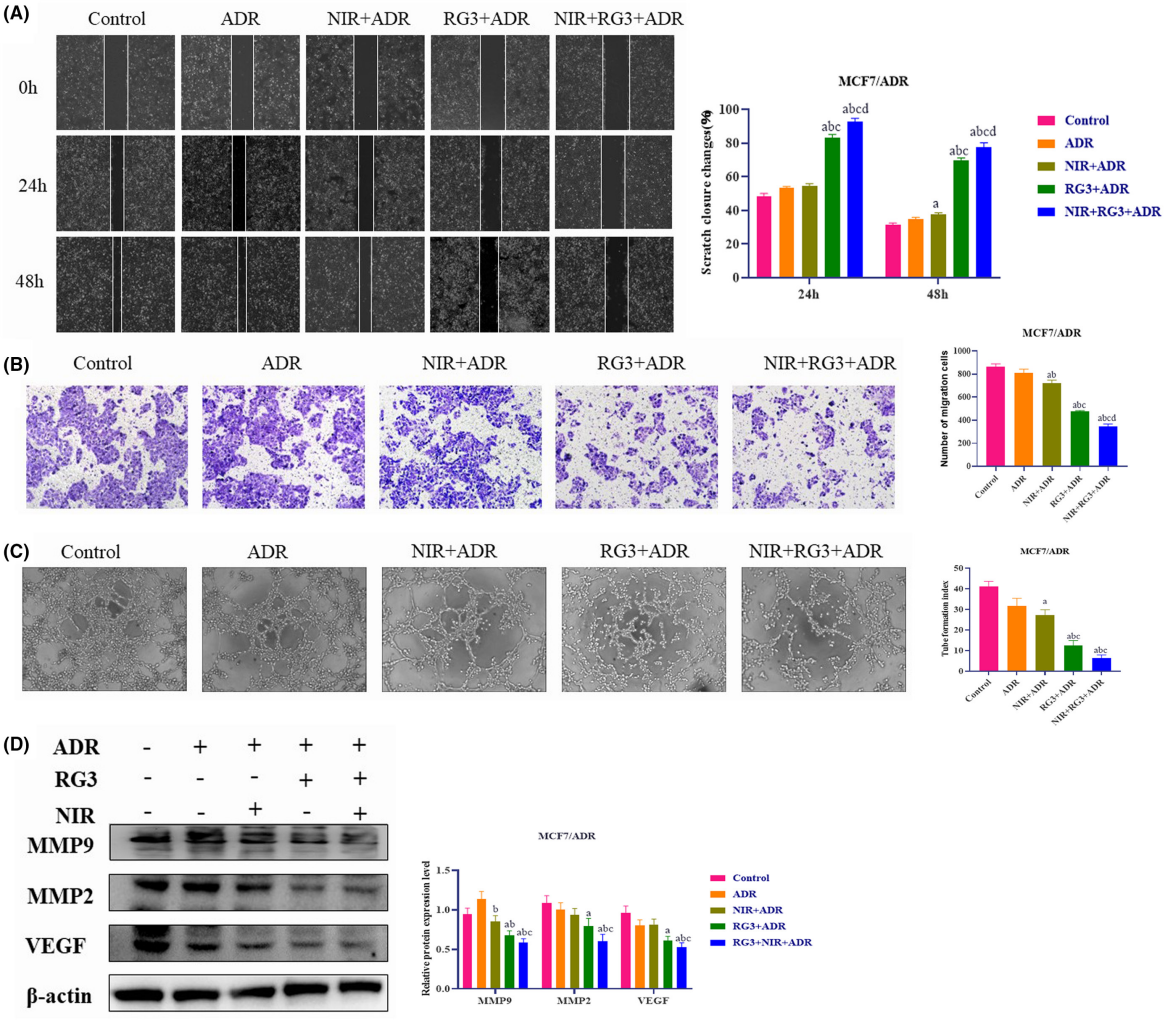
Near‐infrared photothermal therapy combined with ginsenoside Rg3 inhibits migration of MCF‐7/ADR breast cancer cells. (A) Effects of expression levels of different treatment groups on wound healing assays of MCF‐7/ADR cells (100×). (B) Effects of expression levels of different treatment groups on Transwell migration assay of MCF‐7/ADR cells (200×). (C) The role of different treatment group changes in tube formation assays of MCF‐7/ADR cells (100×). (D) Protein expression levels of MMP2, MMP9, and VEGF in MCF‐7/ADR cancer cells were determined by Western blotting. a: *p* < .01 versus control group, b: *p* < .01 versus ADR group, c: *p* < .01 versus NIR group, d: *p* < .01 versus Rg3 group.

The migration process plays a pivotal role in the development and metastasis of malignant tumors (Wu et al., 2021). Therefore, we conducted further investigations to evaluate the potential antimetastatic activity of the Rg3–NIR groups against MCF‐7/ADR breast cancer. Wound healing and Transwell assays were performed, demonstrating a significant reduction in the migratory capacity of MCF‐7/ADR breast cancer cells in the Rg3–NIR groups (Figure 3A,B). To validate our findings, Western blot analysis was conducted, revealing that the Rg3–NIR groups effectively suppressed the protein expression of matrix metalloproteinase 2 (MMP2) and matrix metalloproteinase 9 (MMP9) in MCF‐7/ADR breast cancer cells (Figure 3D).

In addition to migration, angiogenesis is an essential stage in the development and spread of cancer. To investigate the potential of the Rg3–NIR groups in reversing the angiogenic capacity of MCF‐7/ADR cells, we conducted microtubule formation experiments. The results demonstrated a significant inhibition of microtubule formation capacity in the Rg3–NIR groups (Figure 3C). Western blot analysis revealed a downregulation of the vascular marker VEGF (vascular endothelial growth factor) expression level in MCF‐7/ADR cells treated with the Rg3–NIR groups (Figure 3D). When compared to the control‐treated cells, both the NIR and Rg3 groups exhibited inhibition of microtubule formation capacity in MCF‐7/ADR cells, but this inhibition was more pronounced in the Rg3–NIR treatment groups. Thus, the Rg3–NIR group effectively reverses drug resistance in MCF‐7/ADR cells by inhibiting tumor cell migration and angiogenesis.

### 3.4 Rg3–NIR inhibits EMT progression in MCF‐7/ADR breast cancer

**FIGURE 4:**
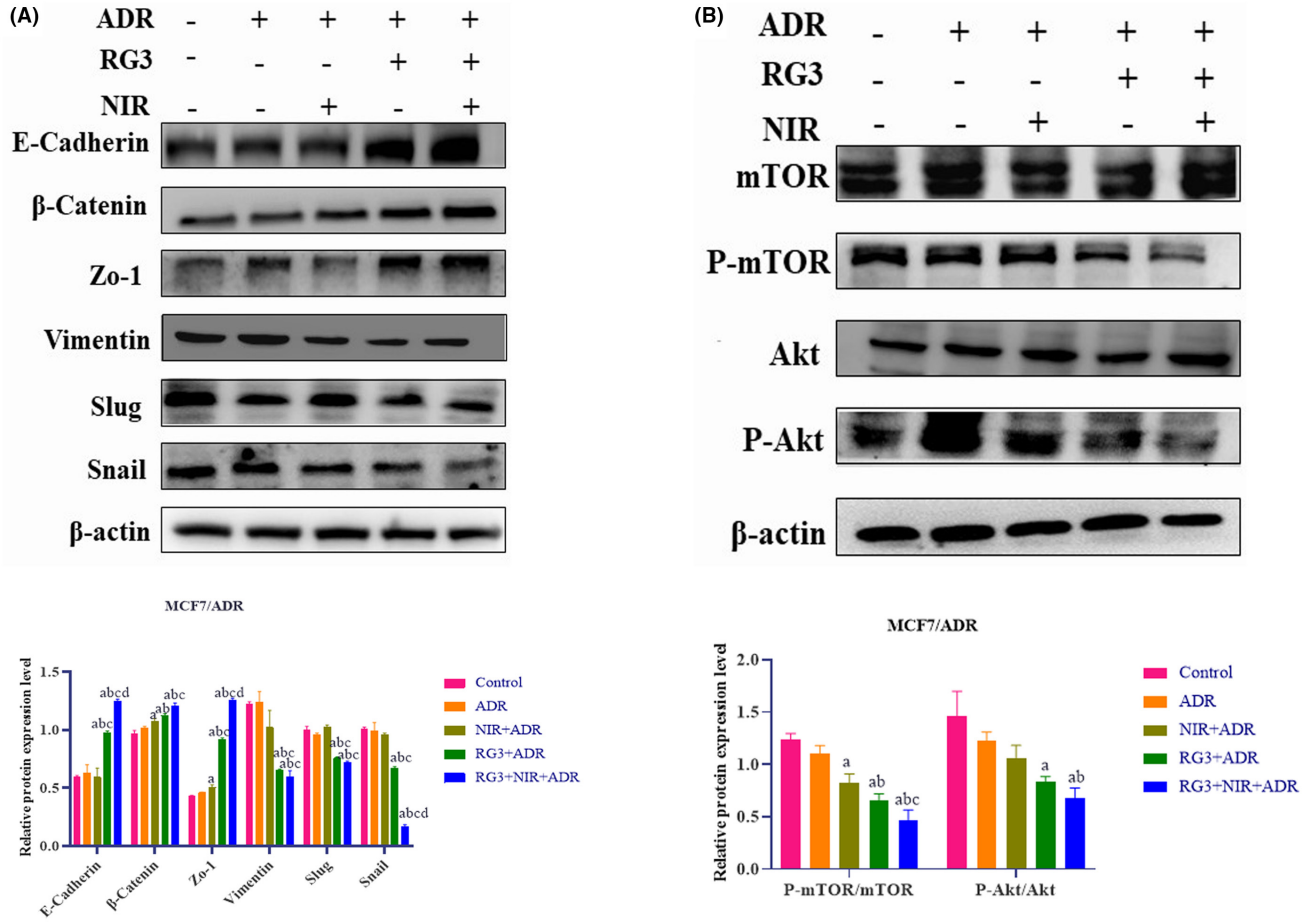
Near‐infrared photothermal therapy combined with ginsenoside Rg3 inhibits EMT labeling and PI3K/AKT signaling pathway in MCF‐7/ADR breast cancer cells. (A) Expression levels of epithelial cell markers and mesenchymal markers in MCF‐7/ADR cells detected by Western blot assay. (B) Western blot detection of the expression levels of PI3K/AKT pathway‐related proteins in MCF‐7/ADR cells. a: *p* < .01 versus control group, b: *p* < .01 versus ADR group, c: *p* < .01 versus NIR group, d: *p* < .01 versus Rg3 group.

Epithelial–mesenchymal transition (EMT) is a biological process characterized by the conversion of malignant epithelial cells into cells exhibiting mesenchymal morphology, which promotes the progression of tumor malignancy. To determine whether the effects of various treatment groups in the EMT process. The alterations in EMT markers were assessed through Western blot analysis (Figure 4A). In comparison with control group, the combined groups of epithelial cell markers E‐cadherin, β‐catenin, and zonula occludens 1 (ZO‐1) were overexpressed, while the mesenchymal markers vimentin, Slug, and Snail displayed low expression. The results showed that the Rg3–NIR group could inhibit the EMT process, thereby reversing the drug resistance of MCF‐7/ADR cells.

### 3.5 Rg3–NIR reverses progression of the MCF‐7/ADR breast cancer through the PI3K/AKT/mTOR (mammalian target of rapamycin) signaling pathway

Recent research indicates that the PI3K/AKT/mTOR signaling pathway is closely linked with the malignant development of tumors. To explore this relationship, we conducted Western blot analysis to assess the levels of mTOR, phosphorylated mammalian target of rapamycin (P‐mTOR), Akt, and phosphorylated Akt (P‐Akt) signaling pathway‐related proteins in MCF‐7/ADR cells (Figure 4B). The results of the Western blot analysis indicate that, when compared with the control group, the Rg3–NIR group demonstrated a significant reduction in the expression levels of P‐mTOR and P‐Akt. This suggests that the Rg3–NIR group may be able to reverse MCF‐7/ADR resistance and breast cancer progression by inhibiting the PI3K/AKT/mTOR signaling pathway.

### 3.6 Rg3–NIR treatment reverses the expression of drug resistance‐related proteins in MCF‐7/ADR cells

**FIGURE 5:**
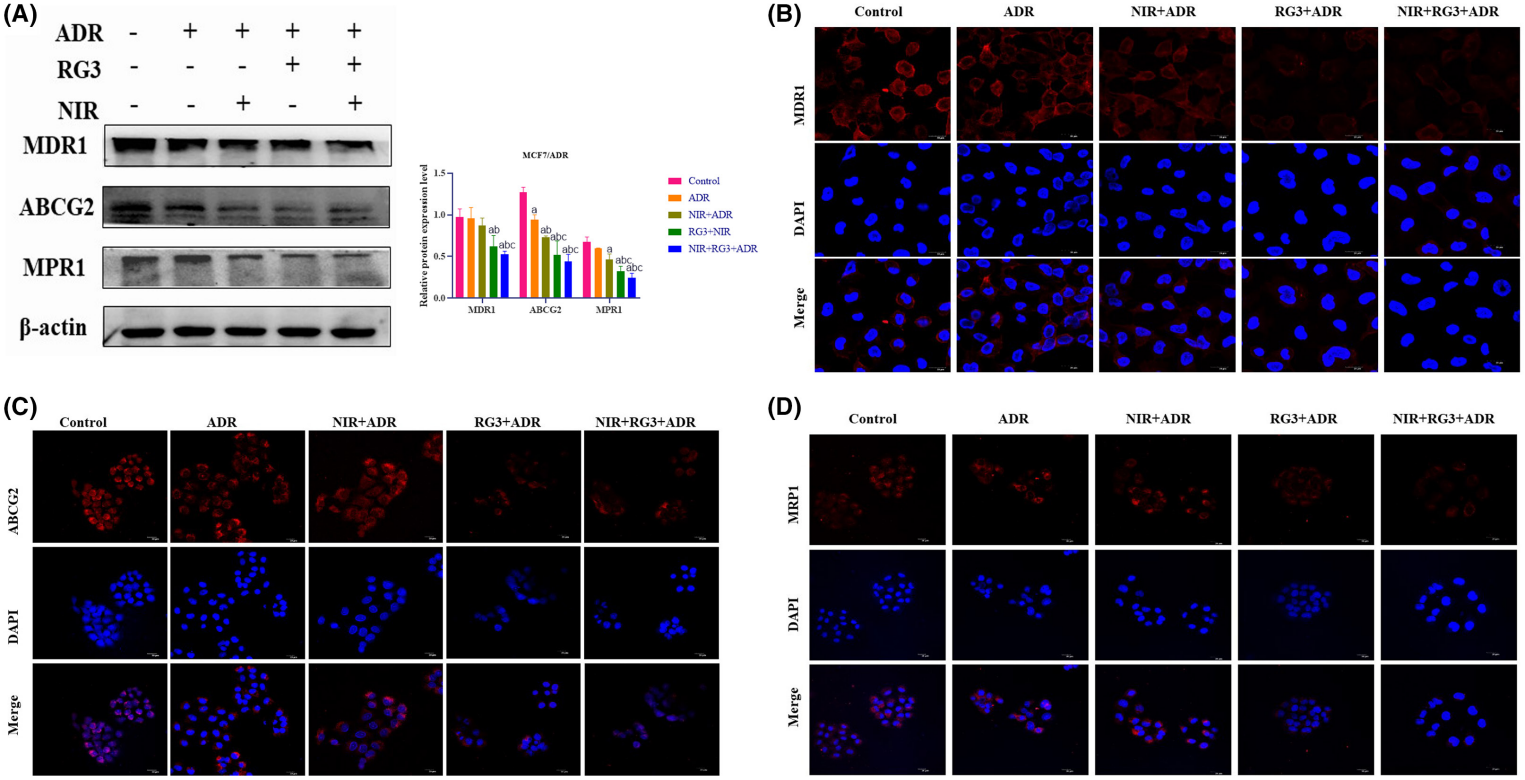
Near‐infrared photothermal therapy combined with ginsenoside Rg3 reverses the expression of drug resistance‐related proteins in MCF‐7/ADR breast cancer cells. (A) Western blot detection of the expression levels of MCF‐7/ADR cell‐related drug resistance proteins. (B‐D) Immunofluorescence detection of the expression of the drug‐resistant protein MDR1, ABCG2, and MRP1 in MCF‐7/ADR cells. a: *p* < .01 versus control group, b: *p* < .01 versus ADR group, c: *p* < .01 versus NIR group.

To delve into the potential reversal of MCF‐7/ADR resistance by infrared light and ginsenoside Rg3, we employed Western blot and immunofluorescence experiments to assess the expression of resistance‐related proteins BCRP and multidrug resistance‐associated protein (MRP) (Figure 5A,D). Remarkably, our findings revealed that both the Rg3 group and the Rg3–NIR group exerted significant inhibition on the expression of MDR1, ABCG2, and multidrug resistance drug protein 1 (MRP1)‐related proteins. These compelling results strongly suggest that a Rg3–NIR can reverse ADR resistance in MCF‐7/ADR cells by effectively inhibiting the expression of resistance‐associated proteins.

### 3.7 In vivo, Rg3–NIR reverses MCF‐7/ADR resistance

**FIGURE 6:**
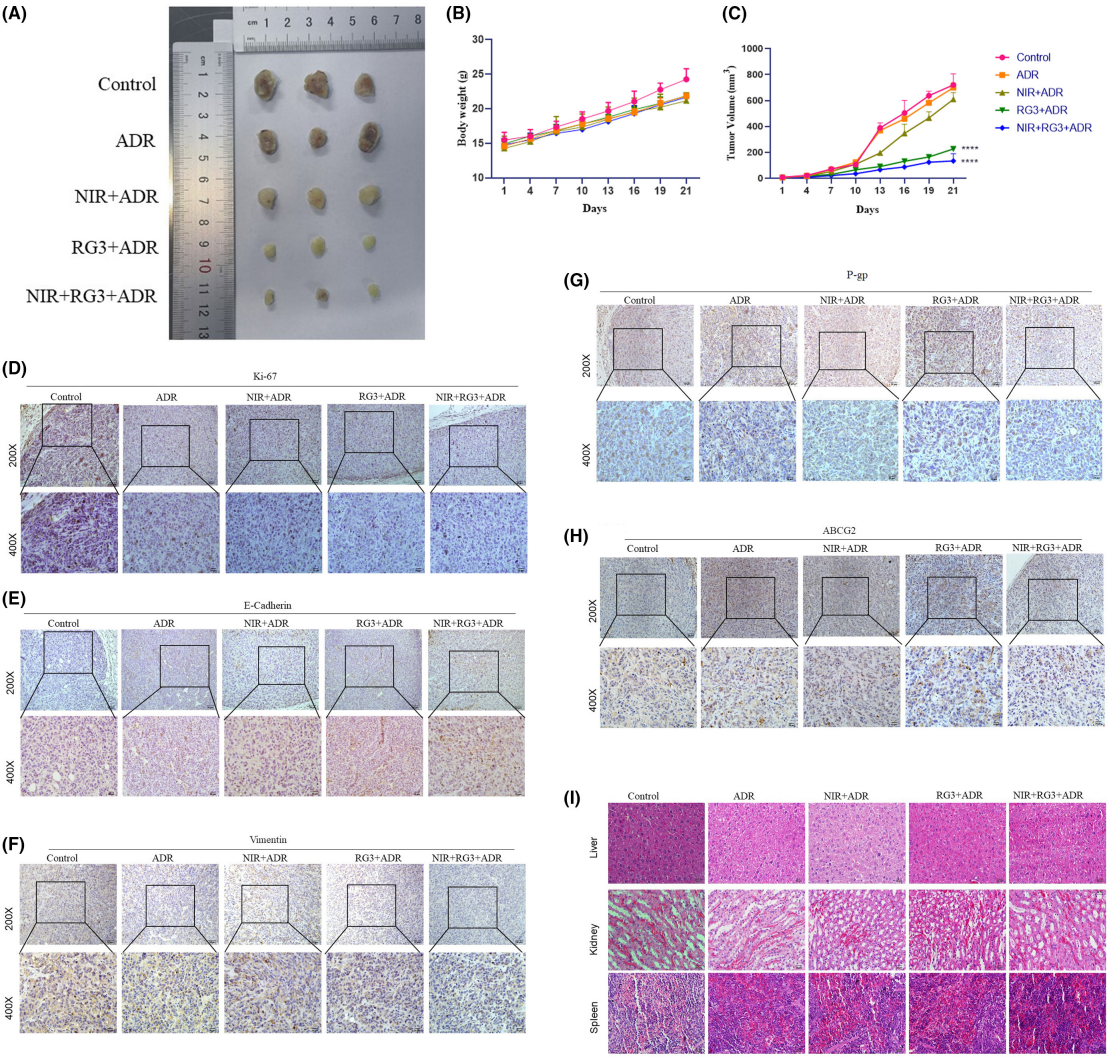
In vivo, near‐infrared photothermal therapy combined with ginsenoside Rg3 reverses MCF‐7/ADR resistance. (A) Tumor growth was monitored for 21 days, tumors were removed and photographed. (B) Body weight of mice. (C) Tumors' size and volume of mice; (D–H) Expression of Ki‐67, E‐cadherin, vimentin, P‐gp, and ABCG2 in tumor tissues after subcutaneous injection detected by immunohistochemistry (IHC). (I) Hematoxylin and eosin (H&E) of liver, kidney, and spleen tissues isolated from each mouse group. ****: *p* < .0001 versus control group.

In order to evaluate the in vivo reversal of drug resistance by Rg3–NIR, MCF‐7/ADR cells were injected subcutaneously to construct a xenograft tumor nude mouse model, the results showed that the tumor volume of both Rg3 and Rg3–NIR groups was reduced compared with the control group, and the reduction of the tumor volume in the Rg3–NIR group was more obvious (Figure 6A,C). Compared with control mice, there was no significant difference in body weight among the NIR,Rg3,and Rg3‐NIR groups of mice (Figure 6B), indicating that there was no physical discomfort to the mice during the experiment. These findings strongly suggest that in an in vivo model, Rg3–NIR therapy can effectively inhibit cancer development. Consistent with the results of in vitro experiments, immunohistochemical analysis showed that Rg3–NIR could effectively inhibit the process of value‐added protein Ki‐67 and EMT transformation (Figure 6D,F). In addition, Rg3–NIR treatment significantly inhibited the expression of drug resistance‐associated proteins P‐gp and ABCG2 (Figure 6G,H). Notably, hematoxylin and eosin (HE) staining showed no significant effects on liver, kidney, and spleen, indicating no adverse effects on these vital organs (Figure 6I). These findings emphasize Rg3–NIR as a promising strategy for reversing chemotherapeutic drug resistance by hindering EMT, reversing the expression of resistance‐associated proteins, and inhibiting cell proliferation, while maintaining a favorable safety profile in vital organs.

## 4 DISCUSSION

Chemotherapy represents the established standard treatment for patients diagnosed with advanced breast cancer (Emens, 2018). Nonetheless, the emergence of resistance to chemotherapeutic agents significantly impacts patient prognosis. P‐gp has been identified as a key contributor to MDR in cancer cells (Abdalla et al., 2021; Li et al., 2017). One of the primary causes of attenuated or loss of efficacy of cancer chemotherapy is the emergence of MDR. It has been shown that multidrug resistance can be overcome by inhibiting or knocking down the dual function of reversing ABC transporter protein‐mediated and AKT‐activation‐enhanced MDR, such as PI3K and P110α (Zhang et al., 2020; Zhang, Yang, et al 2022; Zhang, Li, et al 2022). Consequently, adriamycin, one of the most important antitumor drugs used in breast cancer therapy, is limited in its effectiveness due to being a substrate of P‐gp (Qian et al., 2019). It has been shown that folate receptor‐coupled nanoparticle drugs act as effective drug efflux inhibitors to reverse drug resistance in prostate cancer cells (Singh et al., 2018). Overcoming P‐gp‐mediated resistance remains a significant challenge. We evaluated the therapeutic efficacy of Rg3–NIR as an effective reversal agent in overcoming MDR in MCF‐7/ADR cells. It is urgent to find a way to reduce the adverse reactions of chemotherapy drugs, reverse drug resistance, and not affect their chemotherapy effect. The method is the key to improve the effect of chemotherapy. Our findings demonstrate that Rg3–NIR exerts inhibitory effects on P‐gp function and effectively reverses MDR in MCF‐7/ADR tumor cells expressing P‐gp, both in vitro and in vivo.

Ginsenoside Rg3, a bioactive component derived from ginseng, has been extensively studied for its various pharmacological effects, including immune modulation (Xia, Ma, et al., 2022; Xia, Zhang, et al., 2022), antitumor activity (Hou et al., 2022), reduction of chemotherapy‐induced cardiotoxicity and nephrotoxicity (Sun et al., 2017), reversal of multidrug resistance in tumor cells, as well as protection of the central nervous system (CNS), heart, vascular system, antifatigue, hypoglycemic, and wound healing effects. Ginsenoside Rg3 has shown inhibitory effects on cancer cell growth, induces apoptosis in tumor cells, reversal of abnormal tumor cell differentiation, and inhibition of tumor metastasis (Dai et al., 2019; Yuan et al., 2017). Additionally, near‐infrared photothermal therapy can also influence drug absorption, distribution, metabolism, and excretion, facilitating the entry of chemotherapeutic drugs into cells and altering membrane permeability. Numerous studies have demonstrated that adjuvant therapy combining hyperthermia with chemotherapy benefits patients with recurrent breast cancer (Zhang, Kim, Jin, & Moon, 2017; Zhang, Kim, Jin, Woo, et al., 2017). Near‐infrared photothermal therapy offers an alternative to high‐energy radiation that may damage normal cells, potentially enabling better outcomes in cancer treatment for patients (Li et al., 2021). Based on these properties, our research team proposes to investigate the use of the traditional ginsenoside Rg3 in combination with NIR photothermal therapy to see if it can reverse the drug resistance of MCF‐7/ADR cells and enhance the cytotoxicity of chemotherapeutic drugs.

In order to investigate the impact of near‐infrared photothermal therapy on MCF‐7/ADR cells, we conducted experiments by subjecting the cells to different irradiation temperatures and durations. The drug resistance of MCF‐7/ADR cells to ADR was assessed using the MTT assay. Similarly, the MTT assay was also used to evaluate the effect of ginsenoside Rg3 on MCF‐7/ADR cells, with the EC90 value of Rg3 chosen as the drug concentration.

Some studies have emphasized the role of metastasis in cancer‐related deaths (Fares et al., 2020). In this paper, scratch assay and transwell assay results showed that the Rg3–NIR could significantly inhibit both scratching healing ability and invasion ability. Western blot detection also showed that the Rg3–NIR could inhibit the expression of MMP2 and MMP9.

The key factor in the malignant progression of breast cancer is angiogenesis (Brown et al., 2016). The abnormal formation of vascular network in cancer transports nutrients to tumor cells to promote tumor development and metastasis. Therefore, in order to explore whether the Rg3–NIR affects breast cancer angiogenesis, through the angiogenesis experiment, it was found that the Rg3–NIR could significantly inhibit the ability of lumen formation. Western blot also showed that the Rg3–NIR could inhibit the expressions of VEGF. The results showed its potential to hinder breast cancer cell proliferation by inhibiting luminal formation.

The EMT process plays a critical role in tumorigenesis, invasion, metastasis, and angiogenesis, thereby promoting tumor progression (Pastushenko & Blanpain, 2019). These results indicate that the Rg3–NIR significantly induced the expression of epithelial markers and inhibited the expression of mesenchymal markers in breast cancer cells.

To elucidate the specific mechanism of action of NIR and Rg3 on MCF‐7/ADR cells, we reviewed relevant literature and found that the PI3K/AKT/mTOR signaling pathway plays a crucial role. Next, Western blot experiments demonstrated that the Rg3–NIR significantly inhibited the expressions of P‐mTOR/mTOR and P‐Akt/Akt. Therefore, it can be concluded that the Rg3–NIR inhibits breast cancer progression by targeting the PI3K/AKT/mTOR pathway.

Western blot experiments showed that the Rg3–NIR could reduce the expression of drug resistance‐related proteins BCRP and MDR, indicating that Rg3–NIR could reverse breast cancer drug resistance by reducing the expression of drug resistance‐related proteins.

This study presents a series of experimental findings using Rg3 and NIR to address the malignant characteristics of breast cancer MCF‐7/ADR cells. The results demonstrate that Rg3–NIR treatment inhibits migration, invasion, angiogenesis, and the EMT process of breast cancer cells. It also inhibits breast cancer progression by targeting the PI3K/AKT/mTOR pathway, enhancing the formation and apoptosis rate of breast cancer apoptotic bodies. Furthermore, the Rg3–NIR effectively reverses drug resistance by reducing the expression of drug resistance‐related proteins. However, further research is needed to explore the underlying mechanisms of this drug resistance reversal, including the specific pathways involved and the potential for enhancing the efficacy of adriamycin or reducing chemotherapy dosage through the use of Rg3–NIR. This study aims to provide a novel research approach for the clinical treatment of breast cancer, offering new insights into overcoming multidrug resistance and ultimately improving the overall survival and quality of life for patients battling drug‐resistant breast cancer. This pivotal discovery holds significant implications for the development of innovative therapeutic strategies in the future.

## 5 CONCLUSIONS

In consequence, the Rg3–NIR combination treatment group could inhibit MCF‐7/ADR cell migration, invasion, angiogenesis, and EMT process by inhibiting the PI3K‐AKT–mTOR signaling pathway. It could also reverse the expression of drug resistance‐related proteins, such as ABCG2 and MDR, and promote the increase of apoptosis in MCF‐7/ADR cells, and reverse the drug resistance of MCF‐7/ADR cells.

## AUTHOR CONTRIBUTIONS

**Ying Chang:** Conceptualization (equal); data curation (equal); formal analysis (equal); funding acquisition (equal); investigation (equal); methodology (equal); writing – original draft (equal); writing – review and editing (equal). **Qiang Fu:** Conceptualization (equal); data curation (equal); formal analysis (equal); software (equal); supervision (equal). **Zhongqi Lu:** Investigation (equal); methodology (equal); software (equal); visualization (equal); writing – original draft (equal). **Quanxin Jin:** Conceptualization (equal); data curation (equal); formal analysis (equal). **Tiefeng Jin:** Data curation (equal); formal analysis (equal); methodology (equal); supervision (equal); validation (equal); visualization (equal). **Meihua Zhang:** Funding acquisition (equal); investigation (equal); methodology (equal); project administration (equal); validation (equal); visualization (equal); writing – original draft (equal); writing – review and editing (equal).

## FUNDING INFORMATION

This study received financial support from the National Natural Science Foundation of China: grant number 81960554. This study was supported by a grant from the Jilin Provincial Science and Technology Department Fund: grant numbers YDZJ202201ZYTS179 and YDZJ202301ZYTS131.

## CONFLICT OF INTEREST STATEMENT

The authors declare no conflicts of interest.

## Data Availability

The data underpinning the findings of this study can be obtained from the corresponding author upon request.
